# Clinical significance of radiotherapy before and/or during nivolumab treatment in hepatocellular carcinoma

**DOI:** 10.1002/cam4.2570

**Published:** 2019-10-07

**Authors:** Jeong Il Yu, Su Jin Lee, Jeeyun Lee, Ho Yeong Lim, Seung Woon Paik, Gyu Sang Yoo, Changhoon Choi, Hee Chul Park

**Affiliations:** ^1^ Department of Radiation Oncology Samsung Medical Center Sungkyunkwan University School of Medicine Seoul Korea; ^2^ Department of Medicine Samsung Medical Center Sungkyunkwan University School of Medicine Seoul Korea; ^3^ Division of Hematology‐Oncology Department of Internal Medicine Ewha Womans University College of Medicine Seoul Korea; ^4^ Department of Medical Device Management and Research Samsung Advanced Institute for Health Sciences and Technology Sungkyunkwan University Seoul Republic of Korea

**Keywords:** immunotherapy, liver cancer, nivolumab, radiotherapy, survival

## Abstract

**Background:**

This study aimed to investigate the clinical significance of previous and/or concurrent application of radiotherapy (RT) in the course of nivolumab treatment for advanced hepatocellular carcinoma (HCC).

**Methods:**

Patients with advanced HCC who received nivolumab treatment between March 2017 and May 2018. were included. Nivolumab treatment was indicated in patients who did not respond to conventional therapy including locoregional therapy and/or sorafenib. RT was performed when necessary for curative/palliative purpose.

**Results:**

Among the 76 HCC patients who received nivolumab, 54 (71.1%) had received RT for HCC before and/or during the treatment. The period from initial HCC diagnosis to nivolumab treatment was significantly longer (*P *= .007) and the rate of undergoing transarterial chemoembolization (TACE; *P* = .006) and sorafenib treatment (*P* = .007) was significantly higher in patients who received previous/concurrent RT than in those who did not. Nivolumab‐related toxicities were generally tolerable regardless of the history of RT. During the follow‐up, 39 (51.3%) patients died and 54 (71.1%) patients experienced disease progression according to the RECIST v1.1. Patients who had received previous/concurrent RT had a significantly longer progression‐free survival (PFS; *P* = .008) and overall survival (OS; *P* = .007) than those who did not receive RT; however, this trend was not observed in patients with a history of radiofrequency ablation or TACE (all *P *> .05).

**Conclusion:**

Previous and/or concurrent application of RT in the course of nivolumab treatment was related with longer PFS and OS in advanced HCC patients. Nonetheless, further clinical studies are warranted to confirm our findings.

## Graphical abstract

Nivolumab‐related toxicities in advanced hepatocellular carcinoma were generally tolerable regardless of the history of radiotherapy. Previous and/or concurrent application of radiotherapy in the course of nivolumab treatment was related with longer progression‐free survival and overall survival.

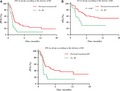

## 1 INTRODUCTION

Hepatocellular carcinoma (HCC) is a major unresolved medical issue as the seventh most common cancer globally and the fourth leading cause of cancer deaths.^1^ Furthermore, HCC is ranked as the second leading cancer globally in regard to absolute years of life lost.^2^ For advanced stage HCC, which accounts for more than two‐thirds of newly diagnosed HCC cases, systemic treatments including sorafenib therapy are recommended based on the positive outcomes of phase III randomized trials.^3^, ^4^, ^5^, ^6^ However, attempts to establish other treatment methods are still ongoing because of unsatisfactory objective response rate of less than 5% and limited time to progression.

With rapid advances in technology and biological research, the application of radiotherapy (RT) for HCC is increasing globally as one of the potent local modalities.^7^, ^8^, ^9^, ^10^ There is growing evidence that transarterial chemoembolization (TACE) followed by RT shows superior outcomes for locally advanced HCC, including portal vein tumor thrombosis, than TACE or sorafenib alone.^9^, ^11^, ^12^, ^13^ On the contrary, prospective trials have shown remarkable effects of immune checkpoint inhibitors (ICI) including nivolumab, as effective systemic agents, in terms of long‐term sustained objective response rate in advanced HCC patients.^14^, ^15^ Based on these outstanding clinical outcomes, nivolumab has been approved and used as one of the valuable therapeutic options for advanced HCC in many countries including Korea.

Substantial data from clinical as well as preclinical studies have continuously suggested the possibility of antitumor immune response enhancement through local RT in combination with ICI treatment.^16^, ^17^, ^18^, ^19^, ^20^ In particular, in the management of non‐small‐cell lung cancer where RT is one of the standard modalities of management and ICI therapy has been increasingly applied recently, prospective studies have confirmed that the combination of these two modalities is associated with good clinical outcomes.^20^

With this background, the authors conducted this study to evaluate the clinical significance of previous and/or concurrent application of RT in the course of nivolumab treatment for advanced HCC.

## 2 PATIENTS AND METHODS

### 2.1 Patients

All patients who received nivolumab treatment between March 2017 and May 2018 at Samsung Medical Center were included in the present study. This study was approved and exempted from obtaining participant consent by the Institutional Review Board of Samsung Medical Center (SMC 2019‐01‐151).

The diagnosis and the management of HCC were proceeded according to the guidelines of the Korean Liver Cancer Association. In the case of special issues, the management policy was decided through a discussion by a multidisciplinary team including a hepatologist, radiologist (diagnostic and intervention), radiation oncologist, and medical oncologist.

### 2.2 Radiotherapy

RT was performed for curative or palliative aims according to the guidelines of the Korean Liver Cancer Association. Liver‐directed RT was performed with respiration gating as previously reported with 10 fractions of three‐dimensional conformal RT (3D‐CRT))/intensity‐modulated RT (IMRT) or three fractions of stereotactic body RT (SBRT).^8^, ^21^, ^22^ Radiation dose of 3D‐CRT/IMRT was determined, as described in a previous article, according to the percentage of the normal liver volume irradiated at more than 50% of the prescribed dose as 5.0, 4.5, 4.0, 3.5, or 3.0 Gy for 10 fractions when 20%‐35%, 35%‐50%, and 50%‐75% of the liver were irradiated, respectively. The eligibility criteria of HCC SBRT were also previously described and were as follows: HCC not suitable for or refractory to surgery and other locoregional modalities such as radiofrequency ablation (RFA) or TACE; HCC 3 cm or less across longest diameter and fewer than three synchronous lesions; adequate residual functional liver volume greater than 700 mL; HCC confined to the liver without extrahepatic metastases; Eastern Cooperative Oncology Group score 0‐1; age ≥20 years; and Child‐Pugh class A or B.

Proton beam therapy (PBT) was performed in selected patients according to the institutional priority policy as described in a previous article. Palliative RT was performed from a single fraction of SBRT to 10 fractions of 3D‐CRT, depending on the status of patients including the extent of disease, liver function, life expectancy, and performance status.^23^, ^24^

### 2.3 Nivolumab treatment

Nivolumab treatment was mainly considered for patients who did not respond to conventional therapy including locoregional therapy and/or sorafenib. During the study period, although nivolumab was approved for its usage in advanced HCC by the Ministry of Food and Drug Safety of Korea, the reimbursement was not covered by the Korean National Insurance Program. Therefore, nivolumab treatment was limited to patients who agreed to pay for the cost of nivolumab. The target patients were planned to receive 3 mg/kg of intravenous nivolumab every two weeks unless disease progression, unacceptable toxicity, or patient refusal made it impossible to continue treatment.^14^ Patients were evaluated at baseline and every three to four cycles of treatment with nivolumab. Adverse events (AEs) were classified according to the National Cancer Institute Common Terminology Criteria for AEs, version 4.0. Other details about nivolumab treatment have been reported previously.^25^

### 2.4 Statistical analysis

To compare the differences in baseline characteristics between the groups classified according to the history of RT, the chi‐square test or Fisher's exact test for categorical variables and the Mann‐Whitney U‐test for continuous variables were used. Overall survival (OS) and progression‐free survival (PFS) were estimated using the Kaplan‐Meier method and calculated as the duration from the starting date of nivolumab treatment to the date when death or progression event were first detected or, to the date of the last follow‐up visit. Progression of the disease was evaluated using the Response Evaluation Criteria In Solid Tumors (RECIST; version 1.1).^26^ The log rank test was used for statistical comparison of the survival curves.

Cox proportional hazards models were used for survival analysis, and for the stepwise selection method, variables with *P* ≤ .1 in the univariable analysis were used in the multivariable analysis. Statistical analysis was performed using SPSS 24.0 software for Windows (SPSS), SAS version 9.4 (SAS Institute), and R 3.4.0 (http://www.R-project.org/). A *P *< .05 was considered statistically significant.

## 3 RESULTS

### 3.1 Patients

During the study period, 76 patients had received at least one cycle of nivolumab for advanced HCC management. Among the 76 patients, 54 (71.1%) had received RT for HCC at least once before (49 patients, 64.5%) or during (12 patients, 15.8%) the nivolumab treatment. Of the 12 patients who had received RT during the nivolumab treatment, seven had also received RT before the nivolumab treatment. The median total sessions of RT in the 54 patients was 1 (range, 1‐8) and the largest fraction size was 5 Gy (range, 3‐22 Gy). Primary liver lesions were irradiated at least once in 35 patients; the remaining 19 patients had received RT for extrahepatic metastatic lesions. Among the 35 patients irradiated for liver lesions, the techniques of RT were SBRT in two, PBT in five, IMRT in seven, and 3D‐CRT in 20 patients. Additionally, nine patients also received SBRT for extrahepatic metastatic lesions. According to the purpose of initial RT, there were nine patients (16.7%) treated with curative intent and the remaining 45 patients with palliative intent.

Baseline characteristics of the patients are shown in Table 1. In the pretreatment evaluation of nivolumab, the Child‐Pugh class of the patients was mainly A, but 14 and three patients showed Child‐Pugh class B and C, respectively. All three patients with Child‐Pugh class C were in the previous/concurrent RT group and patients with Child‐Pugh class B were more frequent in the no RT group. Furthermore, most patients were classified as Barcelona Clinic Liver Cancer stage C. These factors were not different between the groups classified with or without RT before or during nivolumab treatment. In the group of previous/concurrent RT, the period from initial HCC diagnosis to nivolumab treatment was significantly longer (*P* = .007), and the rate of previous history of TACE (*P* = .006) and sorafenib (*P* = .007) was significantly higher than in the group without RT history.

**Table 1:** Baseline characteristics

	History of RT		
	Previous/Concurrent (n = 54)	No (n = 22)	*P* value
Age (years)	62 (37‐81)	64 (40‐82)	.89
Time from initial diagnosis (months)	27.4 (2.0‐190.2)	12.3 (0.5‐119.6)	.007
Sex			
Male	46 (85.2)	19 (86.4)	1.00
Female	8 (14.8)	3 (13.6)	
ECOG performance status			
0	4 (7.4)	2 (9.1)	.76
1	48 (88.9)	19 (86.4)	
2	2 (3.7)	1 (4.5)	
Cause of hepatitis			
HBV	43 (79.6)	13 (59.1)	.12
HCV	4 (7.4)	2 (9.1)	
Alcohol	2 (3.7)	4 (18.2)	
Unknown	5 (9.3)	3 (13.6)	
Child‐Pugh class			
A	45 (83.3)	14 (63.6)	.08
B‐C	9 (16.7)	8 (36.4)	
BCLC stage			
B	1 (1.9)	3 (13.6)	.0
C	50 (92.6)	19 (86.4)	
D	3 (5.6)	0 (0.0)	
Portal vein invasion			
Yes	15 (27.8)	7 (31.8)	.78
No	39 (72.2)	15 (68.2)	
Extrahepatic metastasis			
Yes	45 (83.3)	14 (63.6)	.08
No	9 (16.7)	8 (36.4)	
AFP (ng/mL)	272 (1.3‐193801)	871 (1.3‐200000)	.43
PIVKA‐II (mAU/mL)	2074 (17‐75000)	1071 (16‐75000)	.54
Previous treatment			
Liver transplantation	3 (5.6)	1 (4.5)	1.00
Hepatectomy	28 (51.9)	10 (45.5)	1.00
Metastatectomy	11 (20.4)	2 (9.1)	.33
Radiofrequency ablation	18 (33.3)	3 (13.6)	.10
TACE	43 (79.6)	10 (45.5)	.006
Sorafenib	53 (98.1)	17 (77.3)	.007

Abbreviations: AFP, *α*‐fetoprotein; ALBI, albumin‐bilirubin; BCLC, Barcelona Clinic Liver Cancer; ECOG, Eastern Cooperative Oncology Group; HBV, hepatitis B virus; HCV, hepatitis C virus; PIVKA‐II, protein induced by vitamin K absence‐II; RT, radiotherapy; TACE, transarterial chemoembolization.

The comparison outcomes of baseline characteristics according to the history of RT are shown in Table S1. There was no significant difference in characteristics between patients who underwent concurrent RT plus nivolumab treatment and those who only received nivolumab treatment. The median period between initiation of nivolumab and RT in patients who had received concurrent RT was 1.1 months (range, 0.0‐6.1 months).

### 3.2 Nivolumab treatment and toxicities

Median follow‐up duration of all patients and survivors was 5.7 months (range, 0.5‐22.7), and 12.9 months (2.8‐22.7), respectively. During the follow‐up period, nivolumab treatment was aborted in 62 patients (81.6%) because of disease progression (n = 53), loss to follow‐up (n = 6, 7.9%), liver failure (n = 2, 2.6%), and economic reasons (n = 1, 1.3%). The remaining six patients were lost to follow‐up while the treatment was ongoing. The median duration of nivolumab treatment was 1.5 months (range, 0.0‐19.9 months) with a median four cycles (range, 1‐38). Partial response was observed in nine patients, but no patient showed complete response of disease (objective response rate 11.8%). Among the nine patients who showed partial response, eight had history of previous/concurrent RT and one did not (14.8% vs 4.5%, *P *= .27).

Treatment‐related toxicities were mostly confined to grade 1 or 2, and these improved without any additional management. Table S2 shows the treatment‐related toxicity profile according to the treatment group. There was no noticeable difference in the treatment‐related toxicity for each treatment group.

### 3.3 Survival outcomes

**Figure 1:**
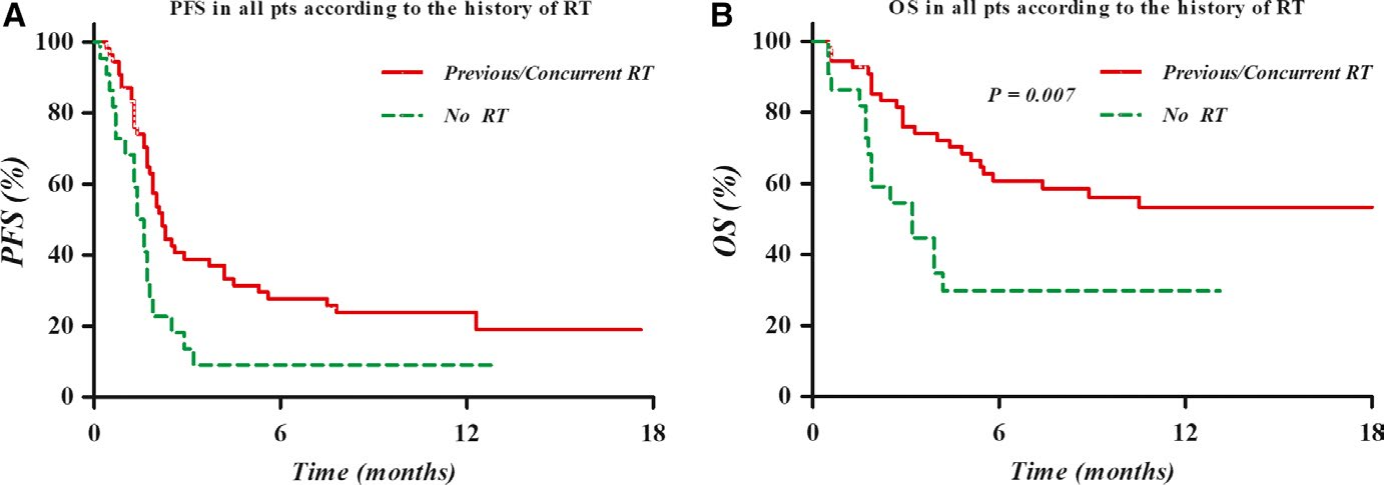
Kaplan‐Meier curve of the progression‐free survival (PFS, A) and overall survival (OS, B) in all patients according to previous and/or concurrent RT. The PFS and OS were significantly higher in patients who had received RT before and/or during nivolumab treatment than in those who had not

During the follow‐up, 39 patients (51.3%) died and 54 (71.1%) experienced disease progression according to the RECIST v1.1. OS and PFS of all patients were 51.8% and 22.0% at 6 months, and 46.2% and 19.1% at 12 months, respectively. As shown in Figure 1, the Kaplan‐Meier curves were significantly different according to the RT combination for both PFS (*P* = .008) and OS (*P* = .007). However, the survival outcomes were no different according to the interval between RT and initiation of nivolumab within 30 and 90 days in the patients who received RT before/during nivolumab treatment (Figure S1). Moreover, the fraction size of RT was not a significant prognostic factor of PFS and OS among these patients, although slightly higher OS rate was noticed in the patients treated with fractions size of higher than 5 Gy (Figure S2). Furthermore, there was no difference in PFS or OS according to target regions of RT (intrahepatic or extrahepatic lesion) (Figure S3).

Additionally, there was no difference between the clinical outcomes and the usage of other locoregional modalities commonly used in HCC management, including RFA and TACE, contrast to the RT (Figure S4).

### 3.4 Prognostic factors for PFS and OS

The outcomes of the univariate and multivariate analyses of the relationship between PFS/OS and probable prognostic factors are summarized in Tables 2 and 3.

**Table 2:** Univariate analysis of probable prognostic factors for PFS and OS

Factors	References	PFS			OS		
		HR	95% CI	*P*‐value	HR	95% CI	*P*‐value
Age > 65 years	Age ≤ 65 years	0.792	0.476‐1.319	.37	0.587	0.305‐1.129	.11
AFP > 200 ng/mL	AFP ≤ 200 ng/mL	1.241	0.744‐2.072	.41	2.345	1.185‐4.641	.01
PIVKA‐II > 1000 mAU/dL	PIVKA‐II ≤ 1000 mAU/dL	1.433	0.848‐2.423	.18	1.709	0.848‐3.443	.13
Male	Female	1.038	0.526‐2.050	.91	1.390	0.612‐3.158	.43
ECOG performance status 2	ECOG performance status 0‐1	1.676	0.668‐4.205	.25	1.770	0.629‐4.983	.17
HBV	Other hepatitis	1.668	0.903‐3.082	.10	1.276	0.605‐2.690	.52
Child‐Pugh B/C	Child‐Pugh A	3.646	2.013‐6.603	<.001	7. 270	3.722‐14.199	<.001
Portal vein invasion (+)	Portal vein invasion (−)	1.093	0.636‐1.877	.75	1.231	0.623‐2.433	.55
Extrahepatic metastasis (+)	Extrahepatic metastasis (−)	0.937	0.515‐1.707	.83	0.536	0.265‐1.084	.08
Liver transplantation (+)	Liver transplantation (−)	0.921	0.288‐2.947	.89	0.963	0.232‐4.000	.96
Hepatectomy (+)	Hepatectomy (−)	1.222	0.740‐2.016	.43	0.865	0.460‐1.625	.65
Metastatectomy (+)	Metastatectomy (−)	0.782	0.407‐1.504	.46	0.739	0.309‐1.766	.50
Radiofrequency ablation (+)	Radiofrequency ablation (−)	0.858	0.485‐1.519	.60	0.678	0.321‐1.430	.31
TACE (+)	TACE (−)	1.084	0.620‐1.895	.78	0.742	0.381‐1.446	.38
Sorafenib (+)	Sorafenib (−)	0.644	0.256‐1.622	.35	0.534	0.187‐1.523	.24
Previous/concurrent RT (+)	Previous/concurrent RT (−)	0.475	0.276‐0.821	.008	0.408	0.212‐0.785	.007

Abbreviations: AFP, *α*‐fetoprotein; ALBI, albumin‐bilirubin; CI, confidence interval; ECOG, Eastern Cooperative Oncology Group; HBV, hepatitis B virus; HR, hazard ratio; OS, overall survival; PFS, progression‐free survival; PIVKA‐II, protein induced by vitamin K absence‐II; RT, radiotherapy; TACE, transarterial chemoembolization.

**Table 3:** Multivariate analysis of probable prognostic factors for PFS and OS

Factors	Reference	PFS			OS		
		HR	95% CI	*P*‐value	HR	95% CI	*P*‐value
AFP > 200 ng/mL	AFP ≤ 200 ng/mL	—	—	—	1.766	0.864‐3.610	.12
Child‐Pugh B/C	Child‐Pugh A	3.340	1.829‐6.098	<.001	6.715	3.382‐13.331	<.001
Extrahepatic metastasis (+)	Extrahepatic metastasis (−)	—	—	—	0.893	0.413‐1.934	.775
Previous/concurrent RT (+)	Previous/concurrent RT (−)	0.541	0.311‐0.941	.03	0.430	0.217‐0.851	.02

Abbreviations: AFP, *α*‐fetoprotein; ALBI, albumin‐bilirubin; CI, confidence interval; HR, hazard ratio; OS, overall survival; PFS, progression‐free survival; RT, radiotherapy.

**Figure 2:**
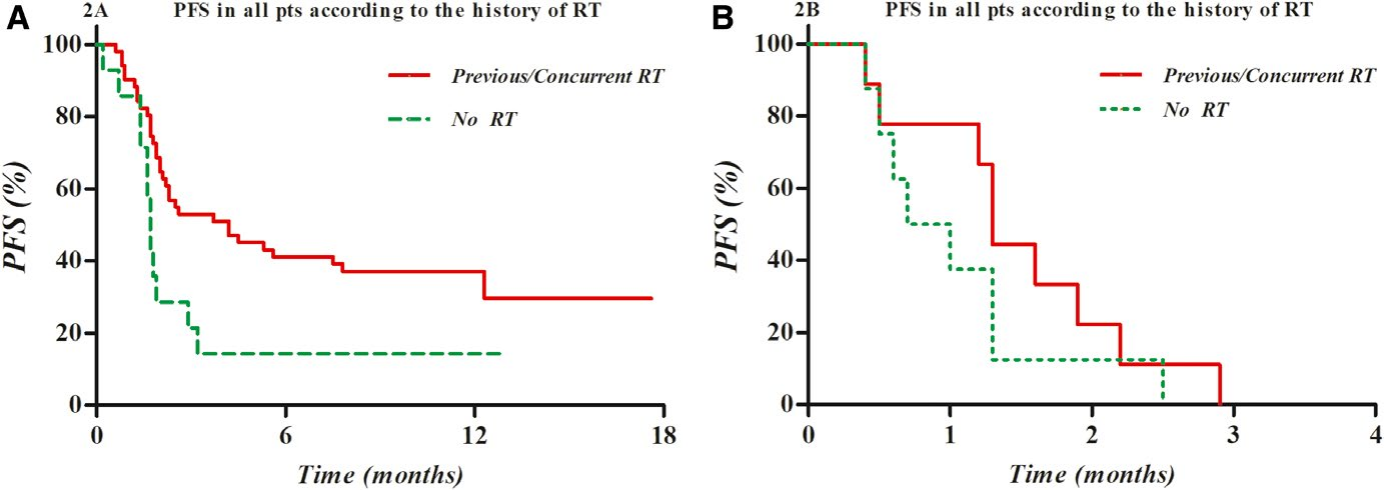
Kaplan‐Meier PFS curves according to the Child‐Pugh class A (A) or B/C (B) with or without RT before and/or during nivolumab treatment: There was a tendency of higher PFS in the group of RT combination before and/or during nivolumab treatment

In the univariate analysis, Child‐Pugh class B or C was a statistically significant poor prognostic factor for PFS (*P* < .001). The only other significant prognostic factor for PFS was the combination of RT before or during nivolumab treatment (*P* = .008). In addition, the significance of Child‐Pugh class (*P* < .001, hazard ration [HR] 3.340, 95% confidence interval [CI] 1.829‐6.908) and the combination of RT (*P* = .03, HR 0.541, 95% CI 0.311‐0.941) as prognostic factors for PFS were similarly noted in the multivariate analysis. The Kaplan‐Meier PFS curves according to Child‐Pugh class and the combination of RT are shown in Figure 2.

**Figure 3:**
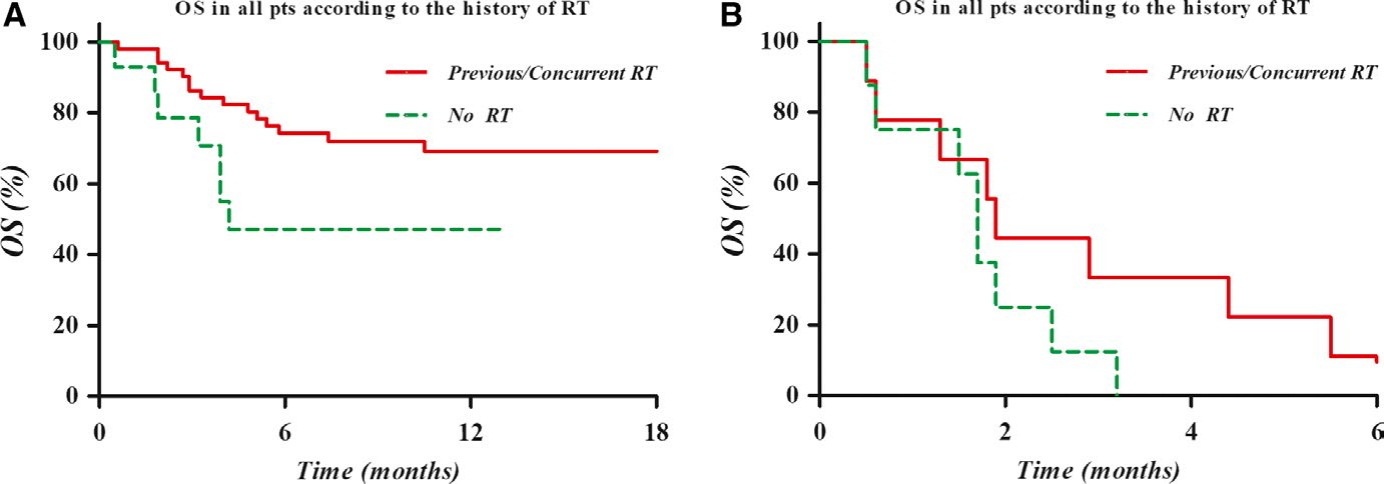
The Kaplan‐Meier OS curves according to the Child‐Pugh class A (A) or B/C (B) with or without RT before and/or during nivolumab treatment: There was a tendency of higher OS in the group of RT combination before and/or during nivolumab treatment

In the univariate analysis of OS, AFP level > 200 ng/mL (*P* = .01) and Child‐Pugh class B or C (*P* < .001) were statistically significant poor prognostic factors. The combination of RT before or during nivolumab treatment was also a statistically significant prognostic factor for OS (*P* = .007). Furthermore, the significance of Child‐Pugh class B or C (*P <* .001, HR 6.715, 95% CI 3.382‐13.331) and the combination of RT (*P* = .02, HR 0.430, 95% CI 0.217‐0.851) as prognostic factors for OS were similarly noted in the multivariate analysis. The Kaplan‐Meier OS curves according to Child‐Pugh class and the combination of RT are shown in Figure 3.

## 4 DISCUSSION

This study evaluated the clinical significance of previous and/or concurrent application of RT in the course of nivolumab treatment for advanced HCC and revealed that patients who received combination treatment had significantly longer PFS and OS than those who did not receive RT before or during nivolumab treatment. Nivolumab treatment showed generally acceptable toxicity profiles, and there was no noticeable difference in treatment‐related toxicities with the use of the RT combination.

Although clinical outcomes of HCC have greatly improved in recent years, it is also true that many unresolved problems remain in the management of the disease. Surgical resection and RFA can be considered firstly as a curative option; however, a limited number of patients are considered candidates for these radical treatment modalities.^27^, ^28^, ^29^ Furthermore, it is well known that recurrence ultimately develops in the majority of patients even after such radical treatments.^30^ Nevertheless, effective adjuvant treatments that can prevent recurrence are yet to be developed. Cases which cannot be resolved by these modalities are treated by TACE in localized disease, and systemic therapies, including sorafenib and lenvatinib, have been recommended.^6^

As the effects of ICI have been reported in prospective trials of several tumors,^31^, ^32^ expectations for its role in the management of HCC have been growing, as it has long been recognized that immune evasion plays a pivotal role in pathogenesis and progression.^33^ Based on promising clinical outcomes, including an objective response rate of 20% and 1‐year OS rate of 62%, in the prospective trial Checkmate‐040,^14^ nivolumab, which is the monoclonal antibody against PD‐1 receptor, has been approved for clinical use in advanced HCC in several countries, including Korea. Nevertheless, it is true that the objective response rate of ICI, including nivolumab (15%‐20%, including a complete response rate of 1%‐2%), remains unsatisfactory in terms of preserving liver function.^14^, ^15^

Compared with the role of RT as a main locoregional modality in other oncologic fields, its use in managing HCC has been very limited because of the radiosensitive nature of cirrhotic non‐tumor liver and the relative radioresistance of HCC. With the rapid advancement in RT techniques and radiation biology, favorable outcomes in prospective studies and the clinical application of liver‐directed RT have been recently increasing.^8^, ^10^, ^13^, ^17^ In fact, several guidelines have recently recommended RT both as a curative modality and a modality with a palliative aim.^27^, ^28^, ^29^

There is growing evidence about the potential synergistic effect of RT and ICI in clinical and preclinical studies.^16^, ^17^, ^18^, ^19^, ^20^, ^32^, ^34^, ^35^ Local RT induces the release of tumor‐specific antigens as in situ individualized tumor vaccines that can result in anticancer immune responses that mediate abscopal effects.^36^ In a prospective trial of non‐small‐cell lung cancer, patients with a history of previous RT had longer PFS and OS with pembrolizumab treatment.^20^

Our findings showed that the clinical outcomes of combination therapy with nivolumab and RT are superior to those of nivolumab alone; these findings are supported by previous preclinical and clinical studies. Furthermore, we found that in advanced stage HCC patients, history of RT before and/or during nivolumab was significantly related with longer PFS and OS. These findings may suggest that an abscopal effect of local RT is associated with ultimate tumor control. Interestingly, in contrast to the use of RT, the use of other local modalities, including surgery, RFA, and TACE, which possibly enhance the release of tumor‐specific antigens and modulate the tumor microenvironment, before nivolumab treatment was not associated with better clinical outcomes.^33^

Those findings suggest that the immune stimulation effect of RT can be differentiated from that of other local modalities based on the nature of RT, that is, slow response, different immune stimulatory signals, and/or modulation of the tumor microenvironment. Moreover, the fraction size of RT and the time interval between RT and nivolumab treatment, which are possibly related to the release of tumor‐specific antigens, were not significantly related to the improvement in survival outcomes. Thus, further prospective trials are warranted to confirm these results and resolve these issues.

This study had several limitations. The study had a selection bias because of its single‐institution retrospective design. In particular, patients with a history of previous RT had significantly a longer duration between HCC diagnosis and nivolumab treatment, implying a relatively low aggressiveness of the tumor itself. Furthermore, there is a possibility that patients receiving RT during nivolumab treatment are limited to good responders of the treatment. In addition, this study presented only the phenomenon without studying the possible relationship between RT on ICI by blood or tissue sample analysis. Therefore, further studies are needed to evaluate the actual role of RT in ICI treatment for advanced HCC. In addition, further studies investigating the relationship between RT and potential biomarkers of ICI response rate, such as the expression of PD‐L1, tissue‐infiltrating lymphocyte, and/or tumor mutation burden, will be necessary.

To our knowledge, this is the first study to evaluate the effect of the use of RT before and/or during nivolumab treatment for advanced HCC, despite the limitations mentioned above. Our findings also confirm the necessity of further research on the synergistic therapeutic effect of combination RT and ICI treatment, which has opened a new horizon in oncologic management. In particular, it is necessary to assess the optimal timing, fraction size, and/or total dose of RT as a combination therapy with ICI treatment.

In conclusion, this study revealed that patients with advanced HCC who received RT before and/or during nivolumab treatment had significantly higher PFS and OS with generally acceptable toxicity profiles. However, further prospective studies are warranted.

## CONFLICTS OF INTEREST

We have no conflict of interest to disclose.

## References

[1] Fitzmaurice C , Akinyemiju TF , Al Lami FH , et al. Global, regional, and national cancer incidence, mortality, years of life lost, years lived with disability, and disability‐adjusted life‐years for 29 cancer groups, 1990 to 2016: a systematic analysis for the global burden of disease study. JAMA Oncol. 2018;4:1553‐1568. 29860482 10.1001/jamaoncol.2018.2706 PMC6248091

[2] Fitzmaurice C , Allen C , Barber RM , et al. Global, regional, and national cancer incidence, mortality, years of life lost, years lived with disability, and disability‐adjusted life‐years for 32 cancer groups, 1990 to 2015: a systematic analysis for the global burden of disease study. JAMA Oncol. 2017;3:524‐548. 27918777 10.1001/jamaoncol.2016.5688 PMC6103527

[3] Cheng A‐L , Kang Y‐K , Chen Z , et al. Efficacy and safety of sorafenib in patients in the Asia‐Pacific region with advanced hepatocellular carcinoma: a phase III randomised, double‐blind, placebo‐controlled trial. Lancet Oncol. 2009;10:25‐34. 19095497 10.1016/S1470-2045(08)70285-7

[4] Forner A , Llovet JM , Bruix J . Hepatocellular carcinoma. Lancet. 2012;379:1245‐1255. 22353262 10.1016/S0140-6736(11)61347-0 PMC13537732

[5] Kudo M , Finn RS , Qin S , et al. Lenvatinib versus sorafenib in first‐line treatment of patients with unresectable hepatocellular carcinoma: a randomised phase 3 non‐inferiority trial. Lancet. 2018;391:1163‐1173. 29433850 10.1016/S0140-6736(18)30207-1

[6] Llovet JM , Ricci S , Mazzaferro V , et al. Sorafenib in advanced hepatocellular carcinoma. N Engl J Med. 2008;359:378‐390. 18650514 10.1056/NEJMoa0708857

[7] Bush DA , Smith JC , Slater JD , et al. Randomized clinical trial comparing proton beam radiation therapy with transarterial chemoembolization for hepatocellular carcinoma: results of an interim analysis. Int J Radiat Oncol Biol Phys. 2016;95:477‐482. 27084661 10.1016/j.ijrobp.2016.02.027

[8] Lee KH , Yu JI , Park HC , et al. Is higher dose always the right answer in stereotactic body radiation therapy for small hepatocellular carcinoma? Radiat Oncol J. 2018;36:129‐138. 29983033 10.3857/roj.2017.00598 PMC6074068

[9] Yoon SM , Ryoo B‐Y , Lee SJ , et al. Efficacy and safety of transarterial chemoembolization plus external beam radiotherapy vs sorafenib in hepatocellular carcinoma with macroscopic vascular invasion: a randomized clinical trial. JAMA Oncol. 2018;4:661‐669. 29543938 10.1001/jamaoncol.2017.5847 PMC5885246

[10] Yu JI , Yoo GS , Cho S , et al. Initial clinical outcomes of proton beam radiotherapy for hepatocellular carcinoma. Radiat Oncol J. 2018;36:25‐34. 29580046 10.3857/roj.2017.00409 PMC5903361

[11] Cho J‐Y , Paik Y‐H , Park HC , et al. The feasibility of combined transcatheter arterial chemoembolization and radiotherapy for advanced hepatocellular carcinoma. Liver Int. 2014;34:795‐801. 24350564 10.1111/liv.12445

[12] Huo YR , Eslick GD . Transcatheter arterial chemoembolization plus radiotherapy compared with chemoembolization alone for hepatocellular carcinoma: a systematic review and meta‐analysis. JAMA Oncol. 2015;1:756‐765. 26182200 10.1001/jamaoncol.2015.2189

[13] Yu JI , Park HC . Radiotherapy as valid modality for hepatocellular carcinoma with portal vein tumor thrombosis. World J Gastroenterol. 2016;22:6851‐6863. 27570422 10.3748/wjg.v22.i30.6851 PMC4974584

[14] El‐Khoueiry AB , Sangro B , Yau T , et al. Nivolumab in patients with advanced hepatocellular carcinoma (CheckMate 040): an open‐label, non‐comparative, phase 1/2 dose escalation and expansion trial. Lancet. 2017;389:2492‐2502. 28434648 10.1016/S0140-6736(17)31046-2 PMC7539326

[15] Zhu AX , Finn RS , Edeline J , et al. Pembrolizumab in patients with advanced hepatocellular carcinoma previously treated with sorafenib (KEYNOTE‐224): a non‐randomised, open‐label phase 2 trial. Lancet Oncol. 2018;19:940‐952. 29875066 10.1016/S1470-2045(18)30351-6

[16] Dovedi SJ , Adlard AL , Lipowska‐Bhalla G , et al. Acquired resistance to fractionated radiotherapy can be overcome by concurrent PD‐L1 blockade. Cancer Res. 2014;74:5458‐5468. 25274032 10.1158/0008-5472.CAN-14-1258

[17] Kim JH , Jenrow KA , Brown SL . Novel biological strategies to enhance the radiation therapeutic ratio. Radiat Oncol J. 2018;36:172‐181. 30309208 10.3857/roj.2018.00332 PMC6226138

[18] Kim KJ , Kim JH , Lee SJ , Lee EJ , Shin EC , Seong J . Radiation improves antitumor effect of immune checkpoint inhibitor in murine hepatocellular carcinoma model. Oncotarget. 2017;8:41242‐41255. 28465485 10.18632/oncotarget.17168 PMC5522235

[19] Postow MA , Callahan MK , Barker CA , et al. Immunologic correlates of the abscopal effect in a patient with melanoma. N Engl J Med. 2012;366:925‐931. 22397654 10.1056/NEJMoa1112824 PMC3345206

[20] Shaverdian N , Lisberg AE , Bornazyan K , et al. Previous radiotherapy and the clinical activity and toxicity of pembrolizumab in the treatment of non‐small‐cell lung cancer: a secondary analysis of the KEYNOTE‐001 phase 1 trial. Lancet Oncol. 2017;18:895‐903. 28551359 10.1016/S1470-2045(17)30380-7 PMC5538772

[21] Yu JI , Kim JS , Park HC , et al. Evaluation of anatomical landmark position differences between respiration‐gated MRI and four‐dimensional CT for radiation therapy in patients with hepatocellular carcinoma. Br J Radiol. 2013;86:20120221. 23239694 10.1259/bjr.20120221 PMC3615390

[22] Yu JI , Park HC , Lim DH , Paik SW . Do biliary complications after hypofractionated radiation therapy in hepatocellular carcinoma matter? Cancer Res Treat. 2016;48:574‐582. 26194367 10.4143/crt.2015.076 PMC4843719

[23] Min JH , Kang TW , Cha DI , et al. Radiofrequency ablation versus surgical resection for multiple HCCs meeting the Milan criteria: propensity score analyses of 10‐year therapeutic outcomes. Clin Radiol. 2018;73:676.e615‐676.e624. 10.1016/j.crad.2018.02.007 29709236

[24] Yoo GS , Park HC , Yu JI , et al. Stereotactic ablative body radiotherapy for spinal metastasis from hepatocellular carcinoma: its oncologic outcomes and risk of vertebral compression fracture. Oncotarget. 2017;8:72860‐72871. 29069831 10.18632/oncotarget.20529 PMC5641174

[25] Lee E , Kim TG , Park HC , et al. Clinical outcomes of stereotactic body radiotherapy for spinal metastases from hepatocellular carcinoma. Radiat Oncol J. 2015;33:217‐225. 26484305 10.3857/roj.2015.33.3.217 PMC4607575

[26] Yoon SE , Hur JY , Lee KK , et al. Real‐world data on nivolumab treatment in Asian patients with advanced hepatocellular carcinoma. Ann Oncol. 2018;29(suppl_8):viii205‐viii270.

[27] Eisenhauer EA , Therasse P , Bogaerts J , et al. New response evaluation criteria in solid tumours: revised RECIST guideline (version 1.1). Eur J Cancer. 2009;45(2):228‐247. 19097774 10.1016/j.ejca.2008.10.026

[28] Korean Liver Cancer . Study Group‐National Cancer Center Korea practice guideline for the management of hepatocellular carcinoma. Korean J Radiol. 2015;16:465‐522. 25995680 10.3348/kjr.2015.16.3.465 PMC4435981

[29] Benson AB 3rd , D'Angelica MI , Abbott DE , et al. Guidelines insights: hepatobiliary cancers, version 1.2017. J Natl Compr Canc Netw. 2017;15:563‐573. 28476736 10.6004/jnccn.2017.0059 PMC5557008

[30] Park HC , Yu JI , Cheng J‐H , et al. Consensus for radiotherapy in hepatocellular carcinoma from The 5th Asia‐Pacific Primary Liver Cancer Expert Meeting (APPLE 2014): current practice and future clinical trials. Liver Cancer. 2016;5:162‐174. 27493892 10.1159/000367766 PMC4960352

[31] Borghaei H , Paz‐Ares L , Horn L , et al. Nivolumab versus docetaxel in advanced nonsquamous non‐small‐cell lung cancer. N Engl J Med. 2015;373:1627‐1639. 26412456 10.1056/NEJMoa1507643 PMC5705936

[32] Wolchok JD , Chiarion‐Sileni V , Gonzalez R , et al. Overall survival with combined nivolumab and ipilimumab in advanced melanoma. N Engl J Med. 2017;377:1345‐1356. 28889792 10.1056/NEJMoa1709684 PMC5706778

[33] Flynn MJ , Sayed AA , Sharma R , Siddique A , Pinato DJ . Challenges and opportunities in the clinical development of immune checkpoint inhibitors for hepatocellular carcinoma. Hepatology. 2019;69(5):2258‐2270. 30382576 10.1002/hep.30337

[34] Demaria S , Golden EB , Formenti SC . Role of local radiation therapy in cancer immunotherapy. JAMA Oncol. 2015;1:1325‐1332. 26270858 10.1001/jamaoncol.2015.2756

[35] Vitale A , Farinati F , Noaro G , et al. Restaging patients with hepatocellular carcinoma before additional treatment decisions: a multicenter cohort study. Hepatology. 2018;68:1232‐1244. 30048016 10.1002/hep.30185

[36] Koller KM , Mackley HB , Liu J , et al. Improved survival and complete response rates in patients with advanced melanoma treated with concurrent ipilimumab and radiotherapy versus ipilimumab alone. Cancer Biol Ther. 2017;18:36‐42. 27905824 10.1080/15384047.2016.1264543 PMC5323007

