# Supplementary material for: Clinical significance of radiotherapy before and/or during nivolumab treatment in hepatocellular carcinoma
Source: Cancer Med. 2019 Oct 7;8(16):6986–94. doi: 10.1002/cam4.2570 (PMC6853810; doi:10.1002/cam4.2570)

**SUPPORTING Table 1.** Comparison of Baseline Characteristics According to the History of RT

|  | Previous RT | | | Upfront nivolumab and concurrent RT | | |
| --- | --- | --- | --- | --- | --- | --- |
|  | Yes (n = 49) | No (n = 27) | *P* -value | Yes (n = 5) | No (n = 22) | *P*-value |
| Age (years) | 62 (37-81) | 64 (40-82) | 0.96 | 61 (56-71) | 64 (40-82) | 0.99 |
| Time from initial diagnosis (months) | 41.5  (2.0-190.2) | 14.9  (0.5-125.7) | 0.004 | 15.0  (6.4-125.7) | 12.3  (0.5-119.6) | 0.61 |
| Sex  Male  Female | 42 (85.7)  7 (14.3) | 23 (85.2)  4 (14.8) | 1.00 | 4 (80.0)  1 (20.0) | 19 (86.4)  3 (13.6) | 1.00 |
| ECOG performance status  0  1  2 | 4 (8.2)  43 (87.8)  2 (4.1) | 2 (7.4)  24 (88.9)  1 (3.7) | 1.00 | 0 (0.0)  5 (100.0)  0 (0.0) | 2 (9.1)  19 (86.4)  1 (4.5) | 1.00 |
| Cause of hepatitis  HBV  HCV  Alcohol  Unknown | 39 (79.6)  4 (8.2)  2 (4.1)  4 (8.2) | 17 (63.0)  2 (7.4)  4 (14.8)  4 (14.8) | 0.27 | 4 (80.0)  0 (0.0)  0 (0.0)  1 (20.0) | 13 (59.1)  2 (9.1)  4 (18.2)  3 (13.6) | 0.87 |
| Child-Pugh Class  A  B-C | 40 (81.6)  9 (18.4) | 19 (70.4)  8 (29.6) | 0.27 | 5 (100.0)  0 (0.0) | 14 (63.6)  48(36.4) | 0.28 |
| BCLC stage  B  C  D | 1 (2.0)  45 (91.8)  3 (6.1) | 3 (11.1)  24 (88.9)  0 (0.0) | 0.11 | 0 (0.0)  5 (100.0)  0 (0.0) | 3 (13.6)  19 (86.4)  0 (0.0) | 1.00 |
| Extrahepatic metastasis  Yes  No | 41 (83.7)  8 (16.3) | 18 (66.7)  9 (33.3) | 0.15 | 4 (80.0)  1 (20.0) | 14 (63.6)  8 (36.4) | 0.64 |
| α-fetoprotein (ng/mL) | 272  (1.3-193801) | 872  (1.3-200000) | 0.27 | 1570  (18.4-34983) | 871  (1.3-200000) | 0.99 |
| PIVKA-II (mAU/mL) | 2315  (17-75000) | 2074  (16-75000) | 0.43 | 2074  (32-44463) | 5069  (16-75000) | 0.78 |
| Previous treatment  Liver transplantation  Hepatectomy  Metastatectomy  Radiofrequency ablation  TACE  Sorafenib  Other systemic therapies | 3 (6.1)  25 (51.0)  11 (22.4)  17 (34.7)  40 (81.6)  48 (98.0)  10 (20.4) | 1 (3.7)  11 (40.7)  2 (7.4)  4 (14.8)  13 (48.1)  22 (81.5)  6 (22.2) | 1.00  0.47  0.12  0.11  0.004  0.02  1.00 | 0 (0.0)  1 (20.0)  0 (0.0)  1 (20.0)  3 (60.0)  5 (100.0)  1 (20.0) | 1 (4.5)  10 (45.5)  2 (9.1)  3 (13.6)  10 (45.5)  17 (77.3)  5 (22.7) | 1.00  0.62  1.00  0.41  0.65  0.55  1.00 |

**SUPPORTING Table 2.** Toxicity Profile According to Treatment Group

|  | Previous/Concurrent RT (n = 54) | | | | No RT (n = 22) | | | |
| --- | --- | --- | --- | --- | --- | --- | --- | --- |
| Event | Gr 1 | Gr 2 | Gr 3 | Gr 2 or 3 (%) | Gr 1 | Gr 2 | Gr 3 | Gr 2 or 3 (%) |
| Skin rash | 2 | - | - | 0.0 | 2 | 1 | - | 4.5 |
| Pruritus | 9 | 1 | - | 1.9 | 1 | 1 | - | 4.5 |
| Fatigue | 3 | - | - | 0.0 | 2 | 1 | - | 4.5 |
| Insomnia | - | - | - | 0.0 | 1 | - | - | 0 |
| Dry cough | 3 | - | - | 0.0 | - | - | - | 0 |
| Mucositis | 1 | - | - | 0.0 | - | - | - | 0 |
| Anorexia | 5 | - | - | 0 | 7 | - | - | 0 |
| Nausea | 4 | 1 | - | 1.9 | 5 | - | - | 0 |
| Diarrhea | 1 | 2 | - | 3.7 | 1 | 2 | - | 9.1 |
| AST elevation | 2 | 2 | 1 | 5.6 | 4 | 1 | 1 | 9.1 |
| ALT elevation | 2 | 2 | 1 | 5.6 | 3 | 0 | 1 | 4.5 |

**SUPPORTING FIGURE 1.** Kaplan–Meier curve of the progression-free survival (PFS) and overall survival (OS) in the patients receive RT before/during nivolumab treatment according to the interval between RT and initiation of nivolumab. The survival outcomes were not different according to the interval between RT and initiation of nivolumab within the 30 (SF 1A, SF 1B) and 90 (SF1 C, SF 1D) days.


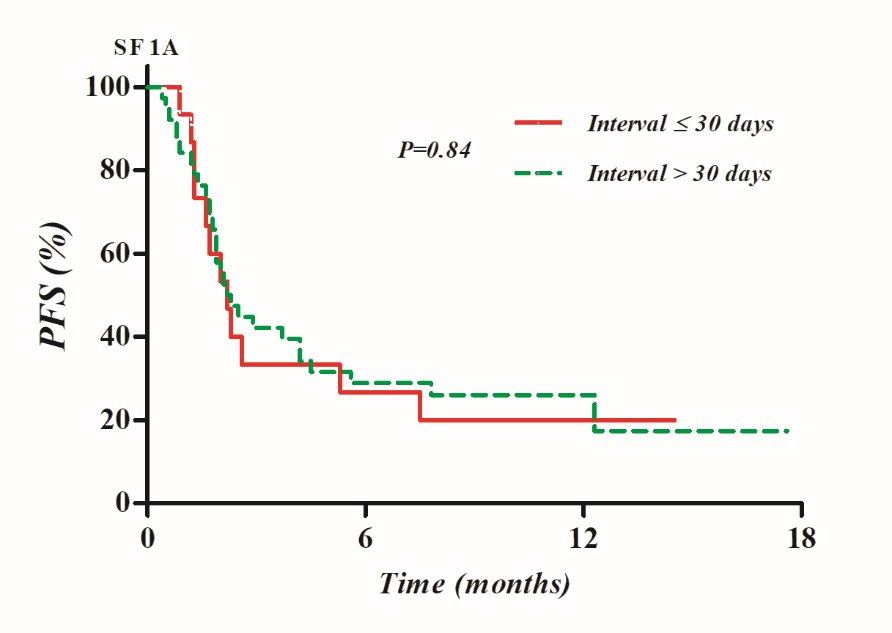


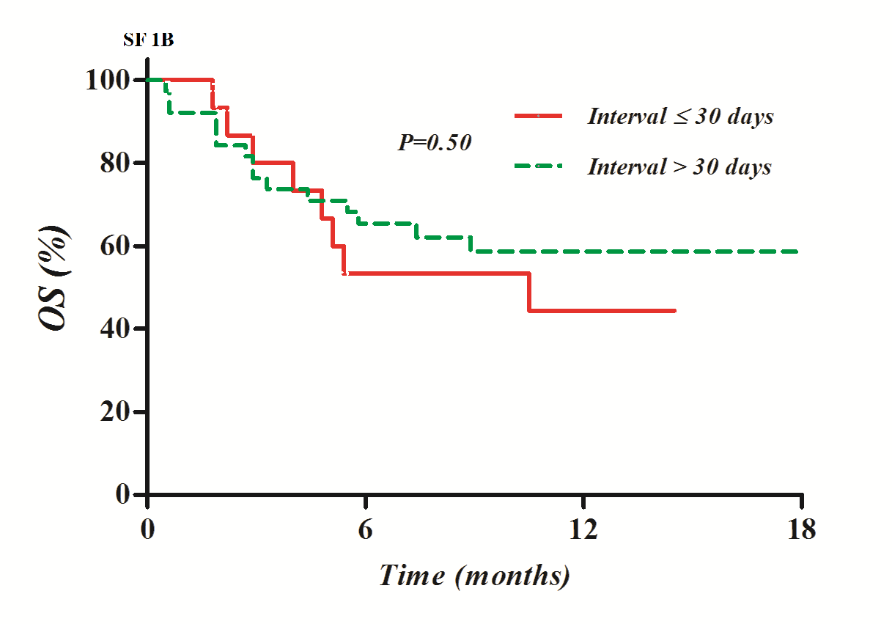


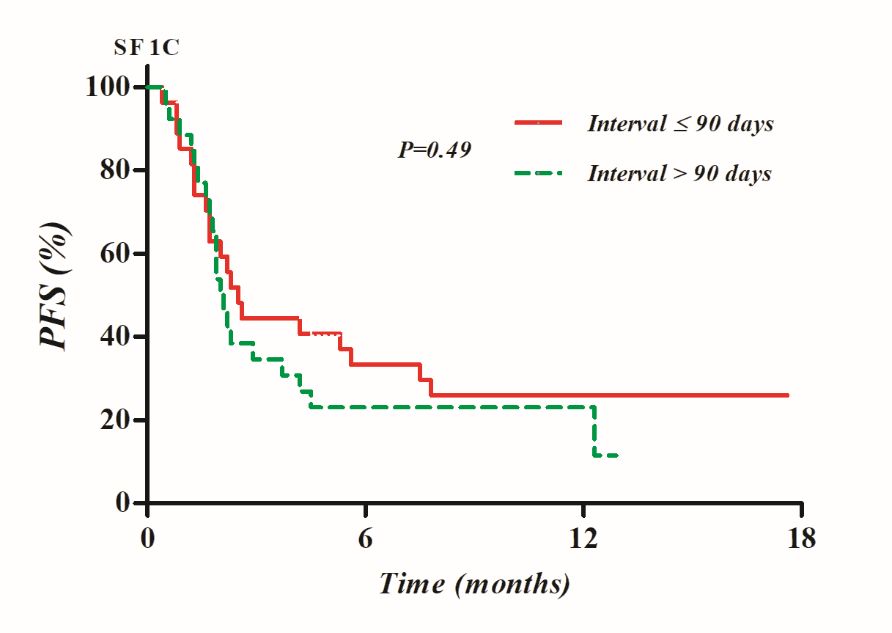


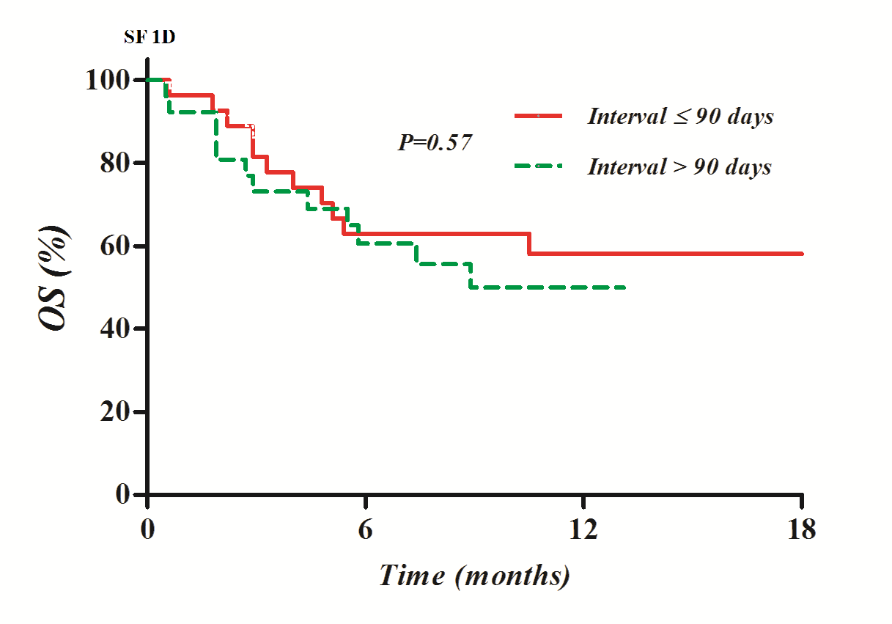


**SUPPORTING FIGURE 2.** Kaplan–Meier curve of the progression-free survival (PFS) and overall survival (OS) in the patients receive RT before/during nivolumab treatment according to the largest fraction size of RT: The survival outcomes were not different according to the largest fraction size of RT (SF 2A, SF 2B), although slightly higher OS rate was noticed in the patients treated with fractions size of higher than 5 Gy (SF 2B).


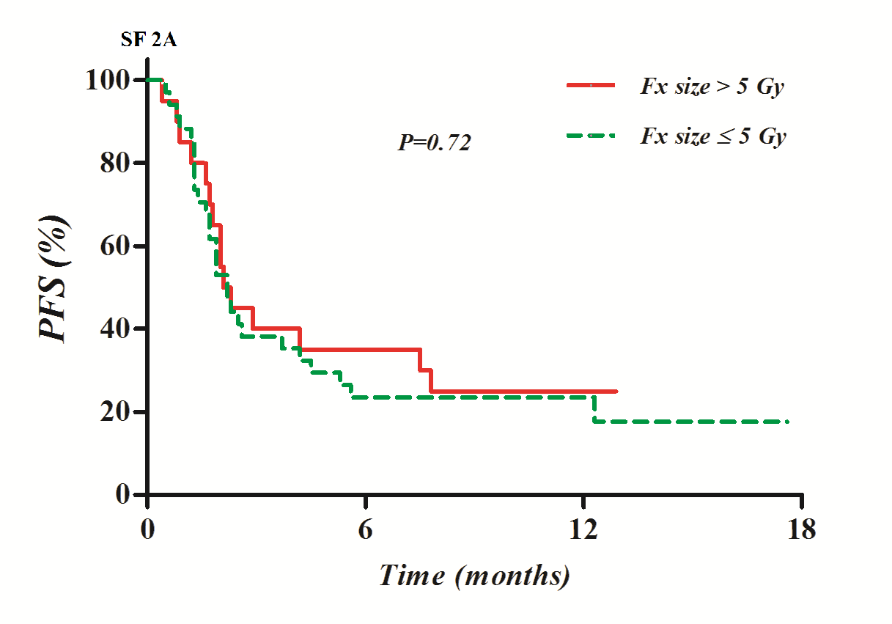


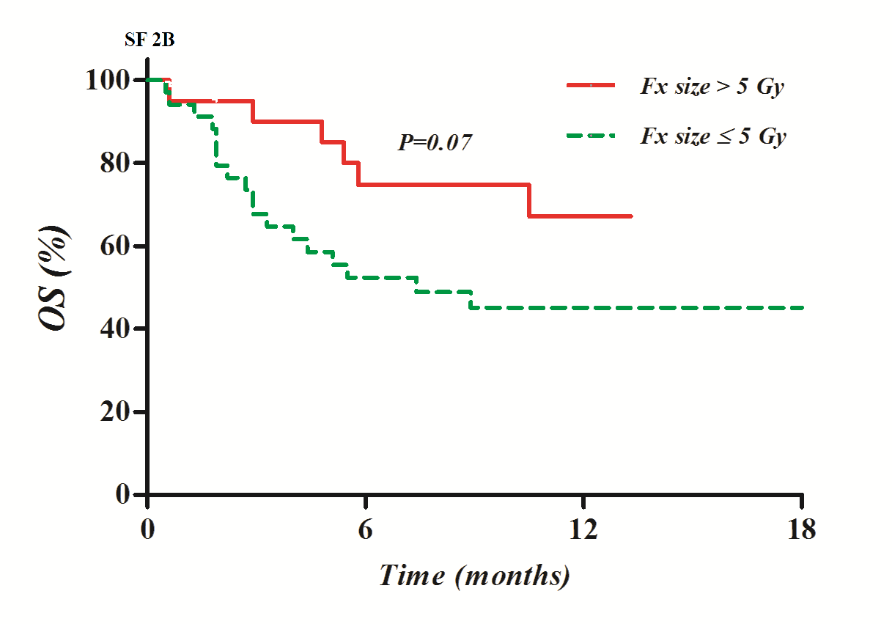


**SUPPORTING FIGURE** 3. Kaplan–Meier curve of the progression-free survival (PFS) and overall survival (OS) in the patients receive RT before/during nivolumab treatment according to the target regions of RT (intrahepatic or extrahepatic lesion): There was no difference in PFS (SF 3A) or OS (SF 3B).


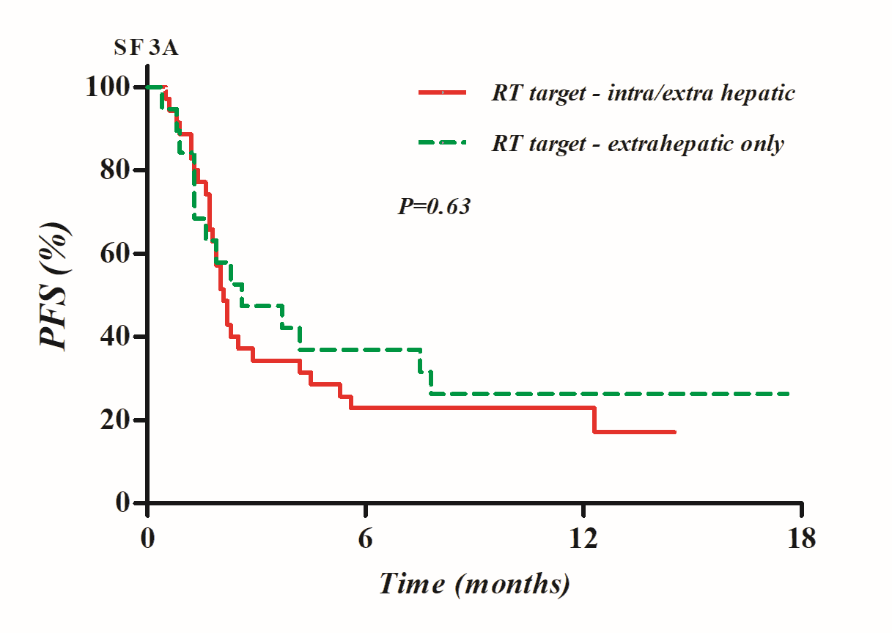


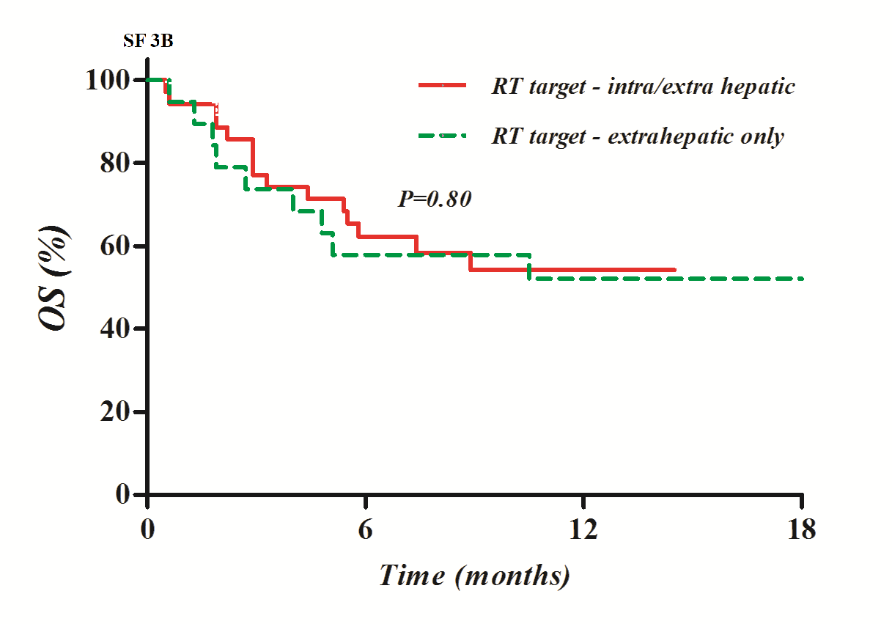


**SUPPORTING FIGURE** 4. Kaplan–Meier curve of the progression-free survival (PFS) and overall survival (OS) in all patients according to previous and/or concurrent RFA or TACE. There was no difference in PFS (SF4A, SF4C) or OS (SF 4B, SF 4D).


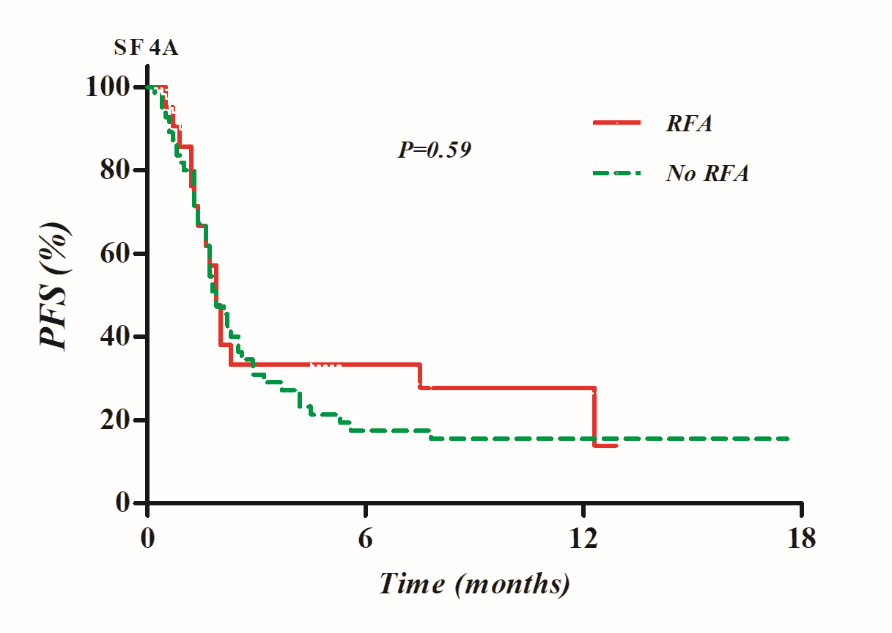


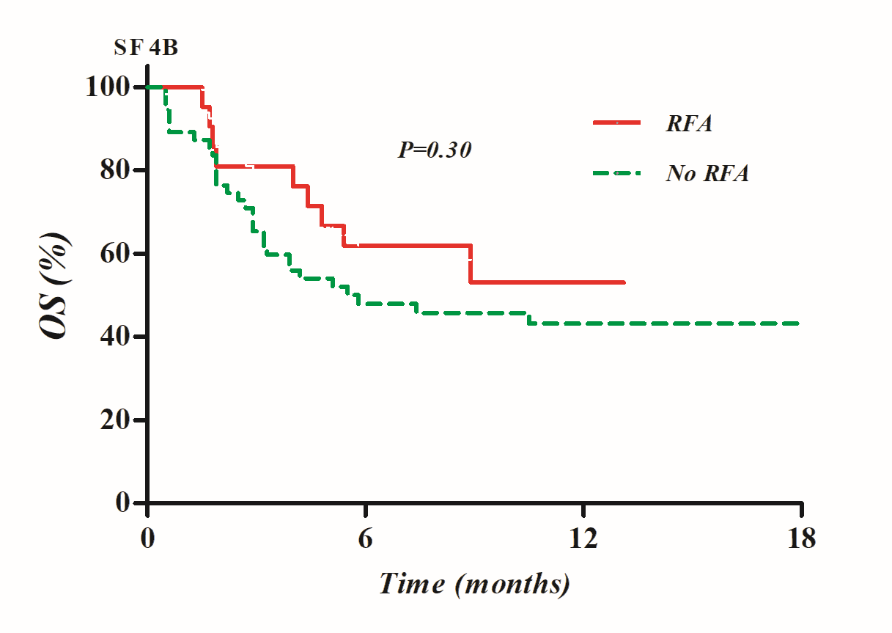


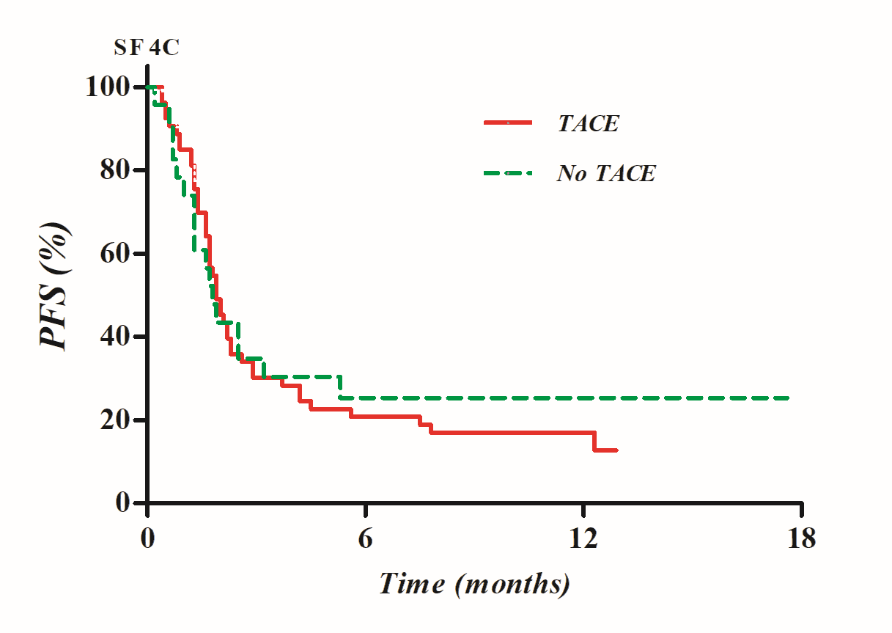


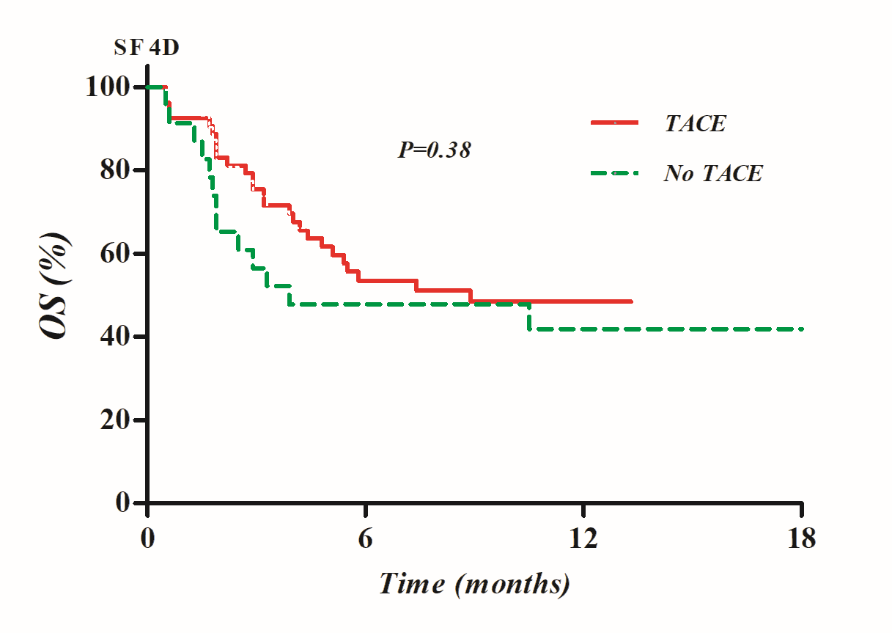

Supplement: Supplementary file 1 [file CAM4-8-6986-s001.docx]
